# An Analysis of Public Opinions Regarding Take-Away Food Safety: A 2015–2018 Case Study on Sina Weibo

**DOI:** 10.3390/foods9040511

**Published:** 2020-04-18

**Authors:** Cen Song, Chunyu Guo, Kyle Hunt, Jun Zhuang

**Affiliations:** 1 School of Economics and Management, China University of Petroleum, Beijing 102249, China; songcen22@126.com (C.S.); 13121188206@163.com (C.G.); 2 Department of Industrial and System Engineering, University at Buffalo, Buffalo, NY 14260, USA; kylehunt@buffalo.edu

**Keywords:** food safety, take-away food, online public opinion, emotional analysis, topic analysis, natural language processing

## Abstract

Take-away food (also referred to as “take-out” food in different regions of the world) is a very convenient and popular dining choice for millions of people. In this article, we collect online textual data regarding “take-away food safety” from Sina Weibo between 2015 and 2018 using the Octopus Collector. After the posts from Sina Weibo were preprocessed, users’ emotions and opinions were analyzed using natural language processing. To our knowledge, little work has studied public opinions regarding take-away food safety. This paper fills this gap by using latent Dirichlet allocation (LDA) and *k*-means to extract and cluster topics from the posts, allowing for the users’ emotions and related opinions to be mined and analyzed. The results of this research are as follows: (1) data analysis showed that the degree of topics have increased over the years, and there are a variety of topics about take-away food safety; (2) emotional analysis showed that 93.8% of the posts were positive; and (3) topic analysis showed that the topic of public discussion is diverse and rich. Our analysis of public opinion on take-away food safety generates insights for government and industry stakeholders to promote the healthy and vigorous development of the food industry.

## 1. Introduction

Food safety issues, such as melamine-contaminated milk powder, heavy metal-contaminated foods, additives which can lead to food poisoning, unhealthy preservatives, and fake foods, pose a great threat to public health [1]. Food scandals have been widely reported and have resulted in social influences [2]. Food safety has become an increasingly important concern in the domestic food market in China over the years, where there is a serious discredit of the food safety systems and official control. Increasing incidents related to food safety have caused consumers to pay more attention to the related quality issues. To some extent, it has led to an overall discredit of the Chinese food market [3].

With an increased consumer demand for convenient services, which results from a more mobile and fast-paced world, many catering industries are beginning to provide take-away food catering services (also referred to as food delivery in some parts of the world). Take-away is very popular among young consumers, such as students and office workers, as it can meet their time-sensitive dining demands. With the rapid development of Internet commerce, the take-away food catering industry is exploding in both consumer demand and revenue, causing great concerns for food safety. Furthermore, various problems with the take-away industry have introduced widespread public concern, such as the case when China Central Television (CCTV) exposed the ordering chaos of Ele.me in 2016 [4]. The issue of food safety supervision in the take-away food industry has received increasing attention. Responding to the frequent occurrence of such phenomena, government agencies have issued laws and regulations for the online food industry (including take-away food) to strengthen the measures that are needed to ensure food safety.

**Figure 1:**
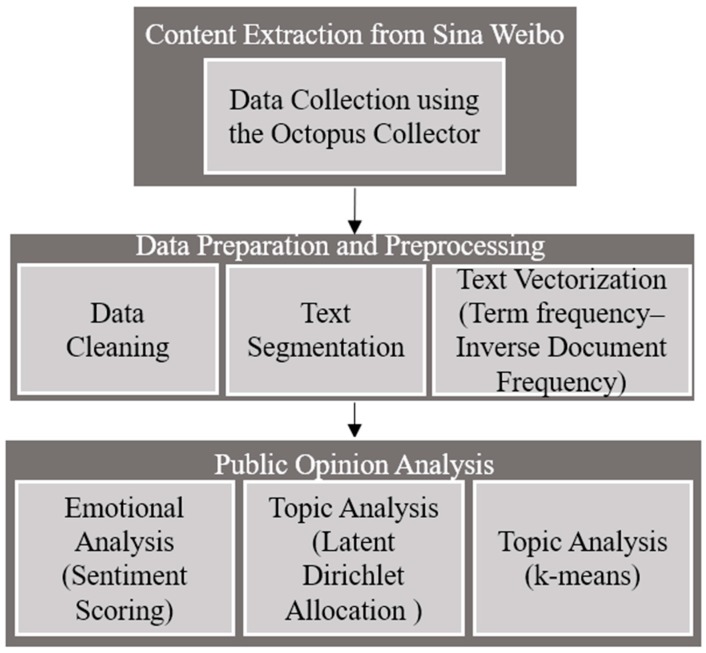
The research framework followed throughout this study.

At present, the chaos in the take-away catering industry has subsided. However, the food safety issue has not been fundamentally rectified, including problems associated with the preparation process and sanitary conditions surrounding take-away foods. Whenever an incident occurs, it leads to a series of hot discussions on online platforms such as Sina Weibo. The discussions on the safety of take-away foods are also multi-faceted, with many people focusing on hygiene and operational practices. For these complicated discussions, traditional data analysis is not enough to study the public’s emotional opinions. To address this, this paper uses an automated learning process to analyze massive online content. The methodologies used throughout this paper are presented in Figure 1.

Previous food safety research has focused on food suppliers [5,6], with very little emphasis on the consumer side. Research on the consumers can be deemed critical to the overall knowledge on food safety in the take-away industry, as consumers drive the demand and are the ultimate decision makers. With a lot of information consumption taking place in the online environment, it can be difficult for consumers to access information related to food processing and kitchen environments. Consumers express their related opinions and emotions on social platforms, such as Weibo.

To the best of our knowledge, research on the evolution of online public opinions relating to the safety of take-away foods is limited. The purpose of this paper is to study the microblogging remarks related to the safety of take-away foods, identify topics of concern, and extract the topic information contained in large amounts of textual data. A dictionary-based sentiment analysis method is used, and emotional trends and tendencies are studied. A public opinion analysis can help government and industry stakeholders to better understand the current situation and provide advice for better food safety regulation in the future.

## 2. Literature Review

### 2.1. Take-Away Food Research

In recent years, the selling of contaminated lunch boxes has frequently occurred, and in general, food safety issues on take-away deliveries are causing global concern. There is no systematic investigation or analysis on the pollution sources, pollution paths, and pollution laws of “secondary pollution” in the food distribution process. From the perspective of both suppliers and consumers, Han et al. [7] gave some suggestions for improving the storage quality of take-away foods, by analyzing the packaging materials for take-away, the various take-out boxes used for distribution, the health status of the distributors, the degree of contact with food, and the factors of delivery time. Yuan and Li [8] proposed an online early warning strategy for food and drug safety incidents from the perspective of improving early warning laws and organizational systems, establishing an online classification monitoring system for food and drug safety incidents, an early warning grading mechanism, and an early warning information release platform.

Scholars have conducted relevant research on the new take-away food industry. Maguire et al. [9] pointed out that take-away food is facing several challenges, such as limited kitchen space, shared cooking equipment, and the short time to complete the orders. Cobb et al. [10] found that between 2014 and 2017, the total number of take-away stores increased by 10% to nearly 58,000, while the proportion of food stores in take-away stores increased by 27%. Adams et al. [11] found through a survey of national diet and nutrition in the UK that 21% of adults and children eat take-away food at home at least once a week, and men eat take-away 5.5% more than women. Keeble et al. [12] showed that the increase in the number of take-away food stores has raised concerns about people’s diet and obesity and proposed to slow down the spread of new stores through an urban planning approach. Take-away food and fast-food consumption continue to grow, which can play an important role in the development and spread of many diseases. Jaworowska et al. [13] examined the nutritional characteristics of take-away food and fast food and reported the link between take-away foods and health. Kirk et al. [14] pointed out that the acquisition and use of take-away foods may be an important determinant of subsequent unhealthy eating behaviors and obesity.

The safety of take-away foods is a social issue that is closely related to people’s lives. Mahon et al. [15] used the planned behavior theory (TPB) to study the attitudes, subjective norms, perceptual control, and habits of British people on ready-to-eat food and take-away food. The results showed that attitude is the most important predictor of food intentions. Further, subjective norms had no effect on take-away foods, and habits were much more important than behavioral intentions. Turrell et al. [16] examined the relationship between individual socio-economic status, regional disadvantages, environmental characteristics of take-away foods, and consumption of take-away foods. It was shown that higher-income and more educated families were more likely to buy take-away food. Timperio et al. [17] discussed the relationship between the availability of a child-supplied take-away store and a consumer take-away store. Miura and Turrell [18] explained the role of take-away food consumption in socio-economic status and body mass index.

### 2.2. Public Opinion Research

Internet public opinion refers to the sum of various emotions, attitudes, and opinions held by netizens in public affairs, especially on hot and trending topics. As a typical interdisciplinary subject, online public opinion research has become the focus of experts in various fields. Savigny [19] showed that in contemporary society, public opinion is usually spread by the mass media. Sobkowicz et al. [20] mined and modeled social media online opinions, where a framework based on social media content analysis and social physics system modeling was proposed for the question of how network opinions are generated, disseminated, and gained. Wang and Zhuang [21] analyzed the content of Hurricane Sandy on Twitter and explored the dissemination of relevant crisis information by the official agencies and the public. Gu et al. [22] proposed that with the development of computer science and technology, the Internet has gradually entered all aspects of society. The popularity of various social software and the development of online media have greatly increased the speed of information transmission and have formed a network of public opinion events. Li et al. [23] designed an adaptive food safety Internet hotspot identification and collection method, that effectively realized the discovery and tracking of public opinions related to food safety. AlSumait et al. [24] introduced an online topic model, that can automatically capture the thematic patterns and identify emerging topics in online content and the related changes over time. The dynamic nature of the proposed method also provides an effective means of tracking topics over time and detecting emerging topics in real time. Tan et al. [25] argued that food safety incidents are typical public safety events and proposed a method to obtain the opinions of netizens from online posts to model food safety incidents.

## 3. Data Collection and Preprocessing of Sina Weibo Posts

### 3.1. Data Collection

This paper uses Octopus Collector to obtain a large dataset about take-away food safety from the Sina Weibo platform between 1 January 2015 and 31 December 2018 [26]. We chose this timeline as a result of the growing popularity of take-away food throughout the recent years, and also the many new food safety laws and guidelines that were issued during this period in China (e.g., Food Safety Law (2015) and Food Safety Supervision and Management Measures for Internet Catering Services (2017)). The data in this study contained Chinese postings published in Sina Weibo, and we directly translated the data into English (i.e., no software was used or needed). The keywords used to extract the Weibo posts include “take-away food”, “food safety”, and “food delivery”. During data processing and cleaning, many posts were deleted due to lack of relevance. Some of these posts consisted of advertisements, while other posts were not related to the topic of this research and were simply noise from the data collection. Some data obtained consisted of pictures and videos, and therefore their content was not appropriate for textual analysis. Therefore, after cleaning the data using these criteria, we were left with a total of 8452 posts that were used for our analysis.

### 3.2. Data Preprocessing

#### 3.2.1. Text Segmentation

Text segmentation refers to dividing a complete and continuous sentence into a set of individual words and punctuation marks, and is a common method used in natural language processing (NLP). In this paper, a word segmentation algorithm based on string matching, also known as a dictionary-based word segmentation algorithm, was used. The algorithm matches text content with the established dictionary according to certain rules in order to perform word segmentation. For example, some original content from the collected Weibo data could be “#Ele.me is unhygienic to eat#.” After text segmentation we get ‘#’, ‘E’, ‘le’, ‘me’, ‘is’, ‘un’, ‘hygienic’, ‘to’, ‘eat’, ‘#’. Note that the website name ‘Ele.me’ would be incorrectly divided into three separate parts. Therefore, we built a customized dictionary and added customized words such as ‘Ele.me’. By using this customized dictionary, the phrase ‘Ele.me’ was treated as a complete and single word for further processing. Table 1 provides a subset of elements that were added to the customized dictionary. Based on the customized dictionary, we do not segment “food safety” as two words due to common sense; this was similar for other words and phrases.

#### 3.2.2. Vectorization of Words

Term frequency–Inverse Document Frequency (TF-IDF), was used to measure the representativeness of words considering the times that the word appeared in a post, and the times it appeared in the corpus of all combined posts. In this paper, we used TF-IDF to construct the word vector matrix. After using TF-IDF and standardizing it, we got a sparse space vector (TF-IDF vector). We then used the word feature vectors of each text to facilitate the subsequent text classification and clustering analyses.

### 3.3. Word Frequency Statistics

After word segmentation of all Weibo content about take-away food safety, word frequency statistics were obtained. The frequency of all words was calculated, and the top 10 words are shown in Table 2.

Table 2 shows that the frequency of words and phrases such as take-away, food safety, Meituan, issue, catering, and other words were high. It becomes clear that consumers in the collected dataset had high interests surrounding the broad topic of take-away food safety.

## 4. Analysis of Online Public Opinion

### 4.1. Dictionary-Based Emotional Analysis of Text

#### 4.1.1. Emotional Analysis Based on Emotional Dictionary

The emotional dictionary, Boson NLP, is based on Weibo, news, forums, and other data sources, and is used to analyze and process textual data that collected from Weibo. The NLP processing is done in Chinese and later translated into English. The Boson NLP emotion dictionary is an emotion polarity dictionary constructed automatically from millions of emotion annotation data, which includes 114,767 words in total. There are many non-emotional words in this emotional dictionary, therefore the actual number of effective emotional words is less than 114,767.

Boson NLP provides a Chinese natural language analysis cloud service that is simple to use, has powerful functionality, and reliable performance. As annotations include microblog data, the dictionary includes many network terms and informal abbreviations, and has a high coverage of non-standard text. The emotion dictionary can be used to build a social media emotion analysis engine, negative content discovery, and other applications. Unstructured data analysis can reveal the trends and correlations hidden in text, and provide strong support for business decision-making, research industry trends, and hot content analysis.

In addition, a word list of negative words (such as nothing, little, few) and a list of adverbs of degree (such as fairly, pretty, rather) are introduced into the processing and are assigned values. We calculate the emotional score of the whole text, instead of defining positive or negative by a single word. The word list of negative words and a list of adverbs of degree are introduced into the processing, and the emotional scores of the posts are obtained. If the score of a post is greater than 0, it is classified as a post with positive emotion, otherwise it is classified as a post with negative emotion. For example, a word segmentation including “Food”, “Food safety”, “Magic formula”, “Take-away”, “APP”, “Qualification”, “Be questioned”, and “0” has an emotional score of 1.72, which means the words show positive emotion.

After emotional classification of all 8452 Weibo posts, the texts with positive emotional value were marked as 1, and the texts with negative emotional value were marked as 0. According to the statistics, there were 7930 emotionally positive posts, accounting for 93.8% of the collected data, while 522 of the posts showed negative emotion. We found that the public’s comments on online take-away food safety were generally positive.

#### 4.1.2. Emotional Time Series Analysis

**Figure 2:**
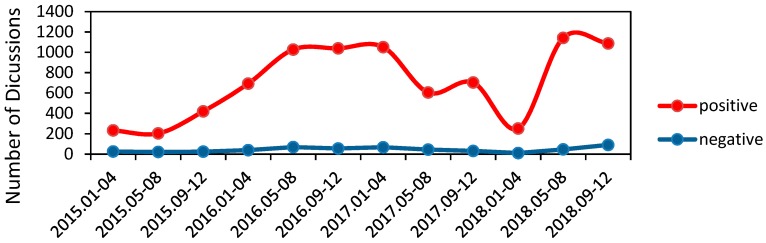
Overall emotional time series.

Figure 2 compares the trends of discussions which have negative and positive emotions. We merge data across four months to draw each point. During these four years, the trends of positive and negative discussions on take-away food safety are similar, although the number of positive posts about food safety are far higher than the number of negative posts.

The number of positive discussions over time has shown an upward trend between January 2015 and May 2016 and between January 2018 and May 2018. It remained at a high level between May 2016 and January 2017 and between May 2018 and September 2018. While between January 2017 and January 2018, the number of positive discussions showed a downward trend. This observation may be due to the times when the FDA (Food and Drug Administration) interviewed the regional agents of Meituan and Ele.me in December 2016 and CCTV exposed black take-away workshops in March 2017. We also observe that the number of negative discussions over time had very little variation.

Table 3 summarizes some of the representative news events in the take-away catering industry between 2015 and 2018. The development of the take-away industry has always been accompanied by governance and rectification, from the new Food Safety Law in 2015, to the “Food Safety Supervision and Management Measures for Internet Catering Services,” which was researched and developed by the State Food and Drug Administration in 2017. These laws have made the take-away industry more transparent and have given the public confidence in the safety of take-away food. In the comparative analysis, most of the public’s emotions were positive.

### 4.2. Topic Analysis

#### 4.2.1. Topic Analysis Based on latent Dirichlet allocation (LDA) Model

Topic analysis was used to determine a text’s topical structure, creating a representation indicating what topics were included in a text and how those topics change within the text. Topic analysis consists of two main tasks: topic identification and text segmentation.

In order to see the trend of food safety of take-away, this paper analyzed the hot words in Weibo posts over four years. Overall, the discussion around the topic of take-away food safety changed over time with the development and progression of society. Using LDA to extract topics, the top 10 hot words associated with each topic are shown in Table 4. In all, we have the following five topics by classification: Topic 1 shows the public’s concern about the take-away industry; Topic 2 concerns college students, mainly reflecting the discussion and influence of this group on take-away; Topic 3 concerns the supervision and management, which shows the public’s attention to the supervision mechanism of the take-away industry; Topic 4 has more objects of concern, such as platforms, journalists, consumers and so on, and demonstrates the wide range of public participation from different users’ perspectives; Topic 5 focuses on the regulatory authorities, such as Food and Drug Administration, Drug Administration, Administration Bureau and so on, such that this topic reflects the public’s concern with the administration and governance of the food safety industry.

Topic extraction of Weibo posts, with respect to different emotional polarities, can be helpful to grasp the different topics that netizens in different emotional states are concerned with. The specific analysis results are shown in Table 5 and Table 6.

Due to the large number of positive emotional posts, this paper divided them into five topics, where each topic was described by up to 10 hot words. Table 5 shows that the topics and the focus of public concern were very similar those in Table 4, which is due to the fact that most of the posts showed positive emotion.

Due to the limited number of negative emotional posts and information, this paper could only divide the posts into three topics, and each topic was described by 10 hot words, as shown in Table 6. Topic 1 relates to college students, and it was obvious that most people held a negative attitude towards the discussion on the delivery time of take-away and whether it could enter schools. Topic 2 show that some consumers were still worried about the take-away catering industry, and there were different worries about businesses and platforms. Topic 3 shows that there were obvious words with strong emotions such as complaint, nausea, and vomiting. It is clear that consumers may have had bad experiences with take-away, such as getting sick, causing them to show negative emotions.

#### 4.2.2. Topic Analysis based on *K*-Means Clustering

*k*-means is an unsupervised machine learning algorithm used to cluster data into a user-defined ‘*k*’ different clusters. This algorithm was selected to analyze the vectorized textual data (which was vectorized via TF-IDF). Through many experiments and analysis, we found that it was better to divide all posts into five topics (*k* = 5), with cluster labels of 0, 1, 2, 3, and 4, respectively. Through the analysis of different topics and mining the similarities, the typical opinions and hot words in each topic are shown in Table 7.

Table 7 shows that hot issues that suddenly appear in society can cause a wave of extensive attention and discussion; e.g., those from black take-away of Meituan and out-of-date products in Baidu. The public often speaks out by criticizing this kind of behavior. As for some of the official policies, the public can also participate in the discussion, and show a positive attitude.

## 5. Discussion and Conclusions

This paper analyzed the public opinion on the issue of take-away food safety by using data mining and Boson NLP techniques. We first collected posts regarding “take-away food safety” from Sina Weibo between 2015 to 2018 using the Octopus Collector. After the posts were preprocessed, users’ emotions and opinions were analyzed using natural language processing and unsupervised learning techniques. This paper fills the research gap by using latent Dirichlet allocation (LDA) and *k*-means to extract and cluster topics from the posts, allowing for the users’ emotions and related opinions to be mined and analyzed.

*k*-means has high computing efficiency, easy operation, and good scalability for big data sets. LDA can find hidden topic information in text via unsupervised learning, and can analyze the topic distribution of the text better. The discussion around the topic of take-away food safety changed over time with various developments. Using LDA to extract topics, there were five topics by classification, which were consistent with the topics and public concerns of positive emotion. Based on *k*-means, it was better to divide all posts into five topics. We found that the results of *k*-means and LDA in terms of topics were similar, as both reflected the public’s attention to the take-away industry and the regulatory mechanisms. However, LDA further demonstrated the distribution table of topics of positive and negative emotion, while there are only three topics due to the limited number of negative emotional posts and information.

Term frequency (TF) was used to show the word frequency statistics, which reflect most people’s concerns better. TF-IDF tends to use less frequent words to prepare for classification. We used TF-IDF to construct the word vector matrix and then use the word feature vectors of each text to facilitate the subsequent text classification and clustering analyses.

The information was quantified and compared with the hot words that identify the focus of users and their emotional trend. Furthermore, our conclusions and suggestions for the industry and research domains are as follows:

(1) Public opinions regarding take-away food safety were studied. Based on the analysis of the relevant data obtained from Sina Weibo during 2015–2018, we conclude that there were many topics about food such as take-away, food safety, businesses, online platforms, and so on. The Online To Offline (O2O) business model perfectly combines online and offline commerce, which has been the developing trend in the take-away food industry. Whether the safety of take-away food is satisfactory or not determines follow-up development of the whole industry.
(2) Users’ emotions and opinions were analyzed using natural language processing. Through the emotional analysis, the proportion of posts exhibiting positive emotions far exceeded that of negative emotions. Due to the timeliness of market supervision and the convenience offered through the services, we conclude that the take-away industry presents an overall positive environment for the consumers.
(3) Representative news events in the take-away catering industry between 2015 and 2018 were summarized. We can see that every step of the development of the take-away industry was accompanied by strict supervision and regulation. Every occurrence of this kind of regulation will cause extensive discussion on microblogging sites. These positive practices make the take-away industry more transparent and ensure the public have increased confidence in the safety of take-away food.
(4) The degree of topics increased over the years, and there were a variety of topics. As for the topic analysis, there were various discussions: some from the regulatory perspectives, some from the policies issued by the relevant departments, and others from different users, such as college students, take-away platforms, and food and drug supervision. For take-away food safety, we conclude that the focus of attention of the public is rich and diverse, therefore the potential management of public opinions should be addressed from multiple different perspectives.
(5) All posts were classed into five topics based on *k*-means clustering. It was found that, on the one hand, people usually showed a negative and bad mood for the bad public opinion events in the society. They thought that the occurrence of such events threatened their daily life, making them have great uncertainty about the future development of the take-away industry, resulting in no confidence in the healthy and safe development of the industry. Therefore, how to build people’s confidence and mutual trust is particularly important in the development of the take-away industry. On the other hand, with a series of policies and measures issued by the relevant departments of the government, great achievements have been made in the cleaning up and rectification of the take-away industry, and a good industry environment has been created. Consumers can really see and feel these changes, therefore public opinion and emotion can move forward in a good direction.

In summary, the government should monitor the public opinion on take-away food safety, pay attention to the opinions of the people, and maintain reasonable guidance and supervision. The measures taken by the Food and Drug Administration and other relevant departments to strengthen the management of take-away platforms and businesses should be continuously strengthened to ensure the health and safety of the general public.

### Limitations and Future Research Directions

There are limitations of this paper, which presents some potential future research directions.

(1) In terms of data selection, this paper crawled relevant data from Sina Weibo, including some contents sent by enterprise or official users. Some of the postings are not significant for emotional analysis, and removing them could improve the accuracy of the emotional analysis.
(2) The emotion dictionary is manually controlled to improve the accuracy of emotion analysis. However, compared with the emotional analysis methods used in machine learning, this method still has some defects such as instability and lack of validation [27]. Future research directions could use deep learning algorithms for further analysis [28].
(3) This study is limited to the opinion of users who posted in China Weibo. The post data from other data source in other countries could result in different results. The geographical distribution of the authors could be further extended and analyzed in different regions in one country or different countries.

## Figures and Tables

**Table 1:** Examples of elements in the user-defined dictionary.

Customized Dictionary	Explanation
Ele.me	A professional online ordering platform in China
McDonald	The world’s largest fast food chain
Food and Drug Administration	Supervise and manage food and drugs in China
315	World Consumer Rights Day
Haidilao	A famous hot pot restaurant
Meituan	Chinese local merchant e-commerce marketplace

**Table 2:** The top 10 high-frequency words.

Hot Word	Frequency	Hot Word	Frequency
Take-away	26,624	Food	8920
Food safety	15,014	Order	6837
Internet	10,688	Issue	5807
Platform	9709	Meituan	5193
Catering	9099	Service	4598

**Table 3:** Relevant News Events.

Date	Relevant News Events
2015.12.9	The revised draft of “The new food safety law” and “The regulations on the implementation of the food safety law” has been solicited from the public.
2016.11.10	The China consumers’ association reported the results of an experiential survey on online take-away ordering services in 2016, showing that food safety problems and the phenomenon of black take-away restaurants still exist.
2016.11.16	Guangzhou launched a special campaign to shut down more than 2000 online food ordering platforms.
2016.11.21	Measures on supervision and administration of food safety for online ordering in Shanxi province were promulgated.
2016.12.27	The Food and Drug Administration (FDA) interviewed Meituan take-away and the regional agents of Ele.me.
2017.3.15	China Central Television (CCTV)’s 315 gala exposes black take-away workshops.
2017.4.15	The general office of the state council issued the 2017 arrangement for key work on food safety, calling for improved judicial interpretation of criminal cases that endanger food safety, and for direct punishment of adulterated and false acts.
2017.12.19	There are more than 20,000 “sunshine catering” stores in Beijing, and 1500 take-away restaurants are broadcast live.
2017.12.27	In order to further strengthen the supervision of online catering, the State Food and Drug Administration worked out the measures for food safety supervision and administration of online catering services.
2018.9.20	Meituan listed on the 20th.
2018.9.26	Haidilao listed on the 26th.
2018.9.19	Starbucks is moving into take-away.

**Table 4:** Topic distribution table.

Topic	Hot Words				
1	Catering	Consumption	Ele.me	China	Internet
	Industry	Deliver	Company	Market	/
2	Student	School	Canteen	University	Campus
	Deliveryman	Health	Work	Worker	Sanitation
3	Network	Catering	Service	Business	Management
	License	Order	Platform	Supervision	/
4	Meituan	Businessman	Consumption	Consumer	Platform
	Meituan Take-away	Discovery	Production	Sanitation	Reporter
5	Platform	Ele.me	Order	Meituan	Network
	Baidu	Food and drug safety	Catering	Food and drug administration	Administration

**Table 5:** Distribution of topics of positive emotion.

Topic	Hot Words				
1	Student	School	Canteen	Deliver	Internet
	University	China	Industry	/	/
2	Meituan	Food and drug administration	Meituan Take-away	Beijing	Drug administration
	Order	Deliveryman	Platform	Network	Beijing
3	Network	Catering	Service	Management	Order
	Platform	Supervise	/	/	/
4	Consumption	Catering	Consumer	Businessman	Entity
	Kitchen	Online	Sanitary	Physical store	Production
5	Platform	Ele.me	Meituan	Baidu	Businessman
	Supervise	Meituan Take-away	Restaurant	/	/

**Table 6:** Distribution of topics of negative emotion.

Topic	Hot Words				
1	Student	School	Canteen	University	Campus
	Time	Prohibit	Classmate	Bad	Sanitation
2	Meituan	Customer	Boss	Platform	Businessman
	Meituan Take-away	Consumption	Canteen	Sanitation	Industry
3	Ele.me	Businessman	Nausea	A	Food
	Discovery	Complain	Hospital	Colleague	Vomiting

**Table 7:** Clustering analysis.

Topic	Typical Weibo Post	High-Frequency Words
1 (234 items)	“Do you dare to eat black take-away?” “Baidu’s own expired food…”	Meituan, help, sorry, outdated
2 (590 items)	“Heavy sound! Deputy director of the FDA: I don’t support home kitchen take-away.” “The person in charge of the online catering service platform made a statement after the collective interview: management measures will be improved.”	Physical store, service provider, food and drug
3 (94 items)	“Ele.me’s first Corporate social responsibility (CSR) report has been released! It is also the first CSR report for the take-away industry. Food safety system upgrade, rider safety protection, support “take-away knight-errant” charity.”	Righteous deeds, rider, security system
4 (1751 items)	““Internet + food ordering” is increasingly popular, but food safety risks. Beijing food and drug administration recently issued a warning online ordering consumption.” “Xinhua criticized the online food delivery platform for its emphasis on expansion rather than supervision, and some shops were not fully licensed for dirty food.” “Several black take-away across the country have been removed from online ordering platforms.”	The third party, Food and Drug Administration, license, service
5 (5783 items)	“Many people participated in the vote launched by People’s Daily online.” “Online To Offline (O2O)food safety issues of food take-away have attracted wide attention and discussion.” “Ele.me and Chinese people’s insurance jointly launch the first food safety insurance policy “take-away insurance”!” “University bans students from ordering take-away…”	Business, catering, food safety, network, consumer, delivery-man, deliver
